# Staged reconstruction of unilateral neglected hip dislocation through total hip arthroplasty and subsequent intramedullary femoral lengthening

**DOI:** 10.1007/s00264-021-05099-x

**Published:** 2021-06-28

**Authors:** Bjoern Vogt, Christoph Theil, Georg Gosheger, Adrien Frommer, Burkhard Moellenbeck, Gregor Toporowski, Robert Roedl, Andrea Laufer

**Affiliations:** 1 Children’s Orthopedics, Deformity Reconstruction and Foot Surgery, University Hospital of Muenster, Muenster, Germany; 2 General Orthopedics and Tumor Orthopedics, University Hospital of Muenster, Muenster, Germany

**Keywords:** Neglected hip dislocation, Developmental dysplasia of the hip, Total hip arthroplasty, Intramedullary lengthening, PRECICE® nail, Subtrochanteric shortening osteotomy

## Abstract

**Background and purpose:**

Total hip arthroplasty (THA) is a successful approach to treat unilateral symptomatic neglected hip dislocation (NHD). However, the extensive leg length discrepancy (LLD) can hereby only be partially corrected. In case of residual LLD of more than 2 cm, subsequent femoral lengthening can be considered.

**Patients/material/methods:**

Retrospective analysis of clinical data and radiographs of five patients (age 38.1 (28–51) years) with unilateral NHD who underwent THA with (*n*  = 3) or without (*n* = 2) subtrochanteric shortening osteotomy (SSO) and secondary intramedullary femoral lengthening through a retrograde magnetically-driven lengthening nail (follow-up 18.4 (15–27) months).

**Results:**

LLD was 51.0 (45–60) mm before and 37.0 (30–45) mm after THA. Delayed bone union at one SSO site healed after revision with autologous bone grafting and plate fixation. Subsequent lengthening led to leg length equalisation in all patients. Complete consolidation was documented in all lengthened segments.

**Conclusion:**

Staged reconstruction via THA and secondary femoral lengthening can successfully be used to reconstruct the hip joint and equalise LLD. The specific anatomical conditions have to be taken into consideration when planning treatment, and patients ought to be closely monitored.

## Introduction

Neglected hip dislocation (NHD) is a condition primarily associated with developmental dysplasia of the hip, but can also be acquired through trauma or septic arthritis of the hip joint [1, 2]. Traumatic as well as septic hip dislocations may, if detected timely, be treated by closed or surgical reduction, even though the rate of avascular necrosis of the femoral head is tremendously high [2–4]. However, if the dislocation has been present for a longer period of time, reduction is often not feasible due to soft tissue contractures and potential nerve damage if reduction is attempted [5].

In children, persistent dislocation results in an inadequate development of the acetabulum and altered bone configuration; frequently, formation of a pseudoacetabulum can be observed [6, 7]. The change in hip biomechanics and the asymmetrical load-bearing within the (pseudo-) acetabulum ultimately leads to secondary osteoarthritis with severe pain and limited mobility [7–9]. In unilateral cases of NHD extensive leg length discrepancy (LLD), which is commonly encountered, may further add to this [10].

Even though technically challenging, total hip arthroplasty (THA) has shown good results in treatment of symptomatic osteoarthritis due to NHD, with satisfying long-term implant survival rates and overall improved quality of life [11–14]. However, complication rates are considerably higher than in primary THA in idiopathic osteoarthritis [13, 15], and LLD can often only be partially equalised through THA. Subtrochanteric shortening osteotomy (SSO) is commonly used to restore the centre of rotation of the hip joint [10, 16]. Furthermore, acute femoral lengthening of more than approximately 4 cm is associated with the risk of nerve palsy [17].

In case of residual LLD of more than 2 cm after THA, subsequent femoral lengthening through distraction osteogenesis may thus be considered to equalise leg length [18].

In recent years, intramedullary lengthening through motorised nails has become increasingly popular, averting complications that are associated with lengthening through an external fixator, such as patient discomfort or pin tract infections with consecutively increased risk for bacteraemia and osteomyelitis [19]. Retrograde femoral lengthening nails can be used for correction of residual LLD after THA [20]. However, data on lengthening procedures performed in femora after THA is scarce [21, 22].

This study investigates complications, radiological results and patient reported outcome following staged THA and subsequent femoral lengthening using a retrograde femoral magnetically-driven intramedullary lengthening nail.

## Patients, material and methods

This is a retrospective single centre analysis that includes patients who were treated for secondary hip osteoarthritis due to unilateral NHD between 2017 and 2020.

After obtaining approval from the local ethical committee (ref. number 2019–368-f-S), a query of the hospital’s electronic database was conducted to identify all patients who underwent staged THA and femoral lengthening using an intramedullary lengthening nail. The indication for THA was symptomatic, advanced osteoarthritis due to NHD in whom previous non-operative treatment had failed. Subsequent femoral lengthening was offered to patients presenting a residual LLD of more than 20 mm after THA who complained of limited mobility due to limping and were dissatisfied with non-operative treatment through shoe lifts. In total, five patients (four female, one male) were identified and included in this study (see Table 1 for patient data).

**Table 1:** Patient data

Case	Sex	Aetiology	Kellgren & Lawrence grade	Crowe grade	Initial LLD (mm)	Age at THA (years)	LLD after THA (mm)	Time THA – PRECICE® (months)	Achieved distraction (mm)	Distraction index (mm/days)	Residual LLD (mm)	Consolidation index (days/cm)
1	female	congenital	n/a	4	60	46	30	9	29	0.97	1	28.6
2	male	septic	4	2	45	36	35	19	34	0.65	1	23.5
3	female	congenital	n/a	4	50	51	45	10	43	0.98	2	23.5
4	female	congenital	n/a	4	50	30	35	7	36	0.97	-1	25.0
5	female	septic	4	2	50	28	40	12	40	1.00	0	24.8

*LLD* Leg length discrepancy, *THA* total hip arthroplasty, *n/a* not applicable

All radiographic planning and measurements were conducted on calibrated radiographs with the PACS® system (GE Healthcare, Chicago, IL, USA) and the post processing software TraumaCad® (Brainlab, Munich, Germany). Prior to any procedure, standing anteroposterior long leg radiographs as well as lateral views of the knee and femur were obtained.

### Surgical technique

A lateral transgluteal approach was used in all patients for THA. In case of severe soft tissue contractures, a SSO was performed to allow positioning of the acetabular implant at the level of the anatomic hip centre. A cementless acetabular cup (Trident® acetabular System PSL, Stryker, Kalamazoo, MI, USA) was used in all cases, combined with a cementless hip stem (DiaLoc®, Implantcast, Buxtehude, Germany) (see Table 2 for implant data). Full weight-bearing was allowed six weeks post-operatively.

**Table 2:** Implant data

Case	Cup diameter (mm)	SSO (mm)	Stem size	Head diameter (mm) / length	Bearing	ILN diameter / stroke / initial length (mm)
1	40	50	-3	28 L	PE – ceramic	10.7 / 50 / 190
2	42	n/a	2	32 S	PE – ceramic	12.5 / 50 / 215
3	42	60	-3	32 XL	PE – metal	12.5 / 50 / 215
4	42	50	-3	32 XL	PE—ceramic	10.7 / 50 / 190
5	40	n/a	1	28 S	PE—metal	12.5 / 50 / 215

*SSO* Subtrochanteric shortening osteotomy, *ILN* intramedullary lengthening nail, *PE* polyethylene, *n/a* not applicable

Subsequent femoral lengthening was achieved through distraction osteogenesis with a retrograde intramedullary lengthening device (PRECICE® (P2.2), NuVasive, San Diego, CA, USA). Weight-bearing of a maximum of 5 kg was permitted. Physiotherapy was initiated during the hospital stay, and patients were educated how to use the external remote control for nail lengthening. Lengthening commenced seven days after implantation, with a distraction rate of 1 mm per day. Clinical and radiological examinations were performed at two-week intervals during the lengthening period, and at six-week intervals during the consolidation period.

### Evaluation and statistical analysis

The following parameters were determined to evaluate the radiological outcome. Kellgren and Lawrence classification and Crowe classification were used to determine the extent of osteoarthritis and dislocation before THA, respectively [23]. The inclination angle of the acetabular cup was measured. The following limb lengthening parameters were evaluated: LLD, distraction index, consolidation index, reliability, accuracy, and precision [19, 24]. The distraction index (length gained per day) was calculated by dividing the total distraction distance through the lengthening time (mm/days) [19]. The consolidation index was measured by dividing the time from the end of distraction to full bone union by the amount of lengthening (days/cm) [19]. The reliability was defined as the ratio of the number of implanted lengthening nails and the number of successfully terminated lengthening procedures with the nail in situ [24]. The accuracy of the intramedullary lengthening system was determined by dividing the distraction distance achieved by the pre-operatively planned distraction distance [19, 24]. The precision was calculated by subtracting the standard deviation of the accuracy from 100 [19].

The functional outcome was determined using the Harris Hip Score, which was obtained before and after THA as well as after distraction at the time of last follow-up.

Descriptive data (mean and range) were reported for the patient cohort. All calculations were made in Microsoft Excel®.

## Results

**Fig. 1:**
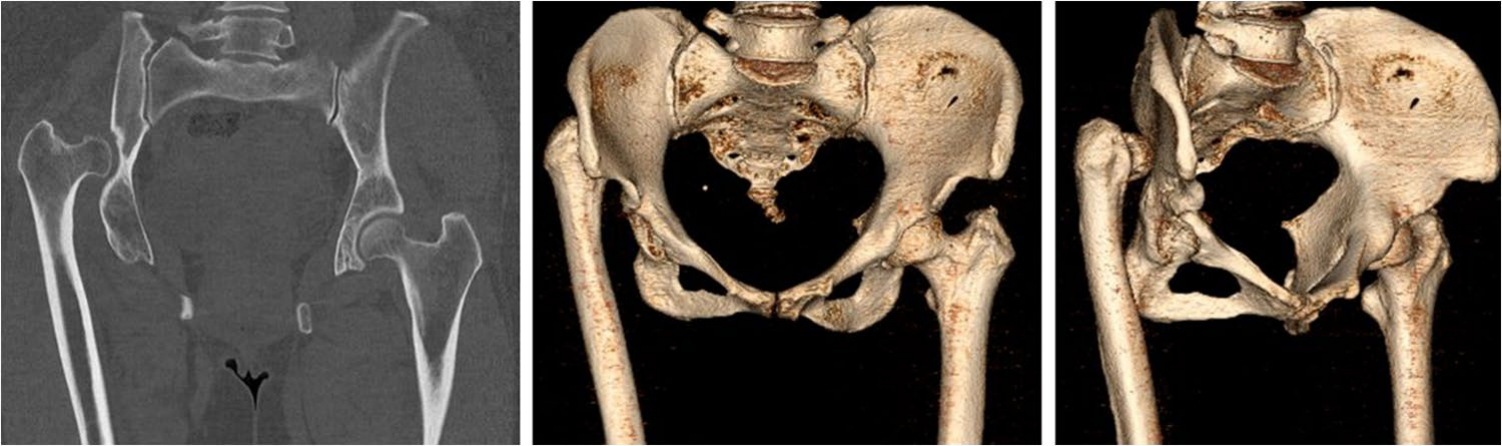
Unilateral congenital high hip dislocation in a 50-year-old woman with dorsoproximal dislocation of the femoral head and formation of a pseudoacetabulum (case 3)

**Fig. 2:**
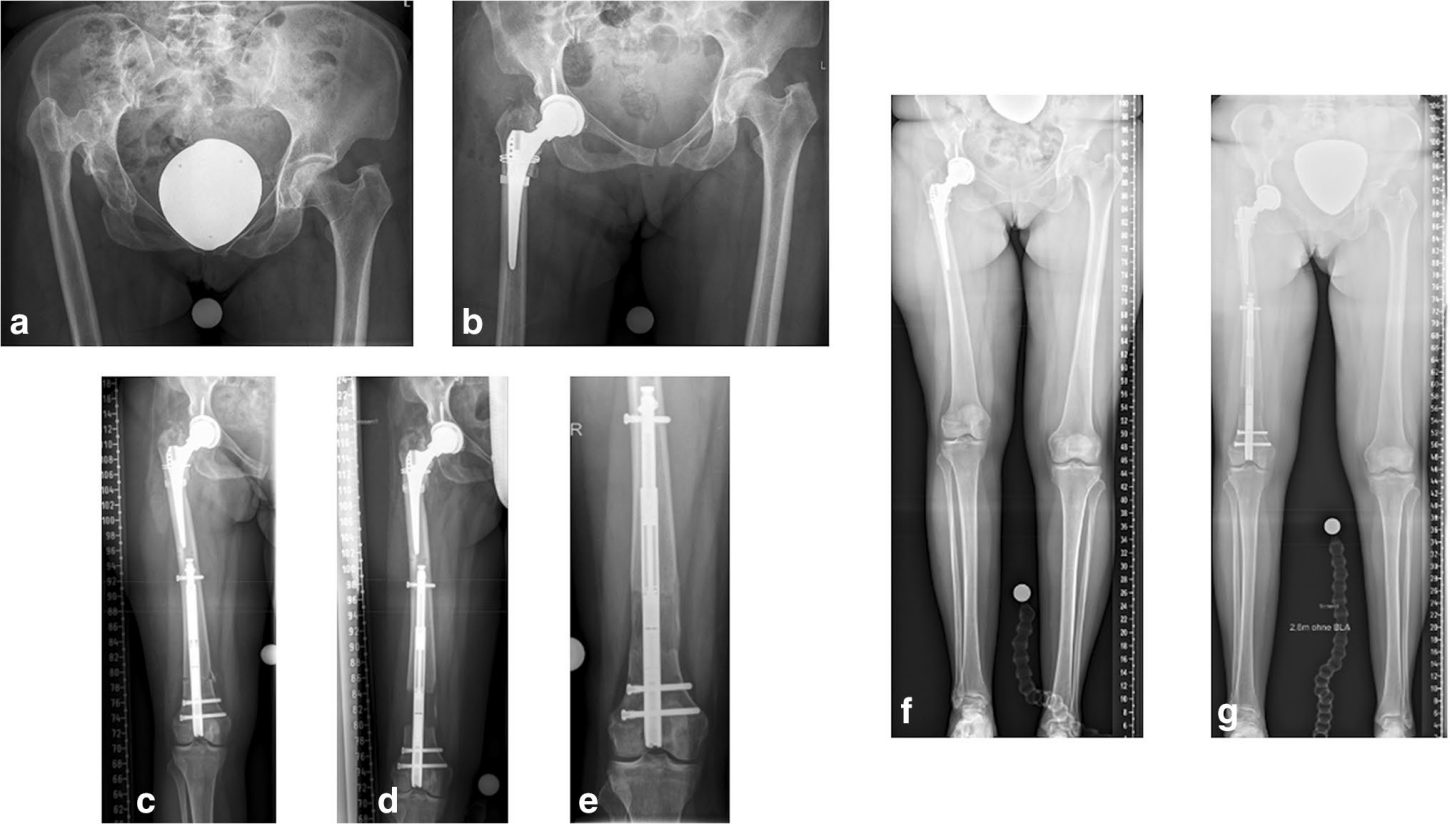
Unilateral congenital hip dislocation in a 50-year-old woman (case 3; see Fig. 1) (**a**). After THA of the right hip (**b**) there was residual LLD of 45 mm (**f**). Femoral lengthening through a retrograde lengthening nail (**c**-**e**) ultimately achieved limb length equalisation (**g**)

**Fig. 3:**
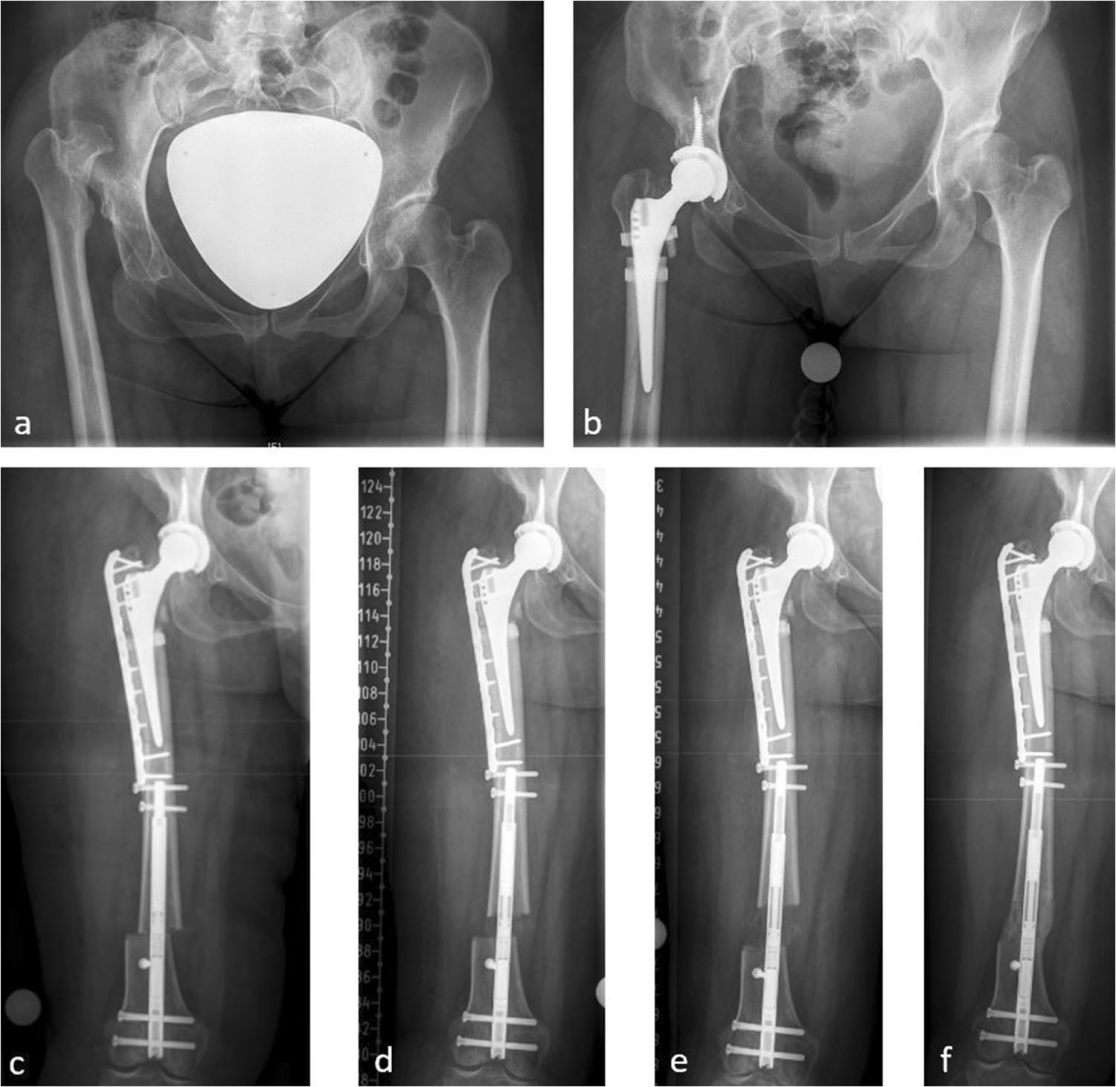
Congenital hip dislocation in a 46-year-old woman (case 1) (**a**). Nine months after THA of the right hip with SSO a pseudarthrosis was observed at the osteotomy site (**b**). Thus, autologous bone grafting from the ipsilateral iliac crest and plate fixation of the proximal femur was performed in addition to implantation of a retrograde lengthening nail (**c**). 29 mm of distraction osteogenesis was achieved over a period of 30 days (**d**, **e**). At the time of last follow-up, eight months after termination of the lengthening procedure, full consolidation at the former pseudarthrosis site and sufficient consolidation at the callotasis site was observed (**f**)

Mean age at first surgery (THA) was 38.1 (28–51) years. Three unilateral NHD were congenital (Fig. 1), and two secondary due to paediatric septic arthritis of the hip joint. Mean pre-operative Harris Hip Score was 47.4 (41.7–53.5). Mean pre-existing LLD was 51.0 (45–60) mm. The femoral head was dislocated 45.5 (15–70) mm proximal. Crowe classification was grade 4 in all patients with congenital NHD, and grade 2 in both patients with NHD due to septic arthritis. SSO of 53.3 (50–60) mm was performed in all three patients with congenital NHD. Mean inclination angle of the acetabular cup was 43.6 (41–48) degrees. Mean residual LLD after THA was 37.0 (30–45) mm. Mean Harris Hip Score improved to 82.7 (67–93) after THA. Retrograde implantation of the intramedullary lengthening nail was performed 11.4 (7–19) months after THA (Fig. 2). In one patient in whom delayed bone union of the SSO site was observed, additionally autologous bone grafting from the ipsilateral iliac crest and plate fixation of the proximal femur was performed (Fig. 3). All lengthening procedures were finished with the nail remaining in situ until the end of distraction showing an excellent reliability of 100%. Mean achieved distraction distance measured 36.4 (29–43) mm resulting in a residual LLD of 0.6 (-1–2) mm after treatment termination. Thus, leg length equalisation was achieved in all patients. Mean distraction time was 40.6 (30–52) days. Mean distraction index was 0.91 (0.67–1.00) mm/d. Accuracy and precision of distraction calculated to 97.3% and 98.5%, respectively. Complete consolidation of all lengthened segments was documented. Mean consolidation index was 25.1 (23.5–28.6) d/cm.

One patient presented 40° flexion deformity of the knee joint after distraction of 20 mm, thus the nail was retracted until full extension was restored and a long-leg plaster cast was applied, within which distraction was resumed.

No hardware complications or infections were observed during or after the distraction period. Mean follow-up after THA was 18.4 (15–27) months. At the time of last follow-up, all patients were able to ambulate without walking aids and free of pain. Mean Harris Hip Score further increased to 95.7 (86.8–100). Range of motion of the knee joint was sufficient in all but one patient, who showed a limited flexion of 100° but was able to fully extend the knee (see Table 3 for functional results). To date, nails were removed in two patients, 12 and 13 months after implantation, respectively.

**Table 3:** Patient data – functional results

Case	Initial HHS	HHS after THA	HHS at last follow-up	Initial ROM of the knee: extension / flexion (°)	ROM of the knee at last follow-up: extension / flexion (°)	Initial ROM of the hip: extension / flexion internal / external rotation abduction / adduction (°)	ROM of the hip at last follow-up: extension / flexion internal / external rotation abduction / adduction (°)
1	42.4	71.8	86.8	0/0/140	0/0/110	0/0/90 30/0/5 60/0/10	0/0/100 30/0/40 40/0/10
2	46.1	93.0	100.0	0/0/130	0/0/120	0/0/90 0/0/0 10/0/20	0/0/110 30/0/30 20/0/20
3	53.4	92.8	99.8	0/0/150	0/0/150	0/0/150 50/0/50 10/0/10	0/0/110 10/0/50 20/0/20
4	53.5	88.8	95.9	0/0/140	0/0/130	0/0/90 40/0/40 40/0/10	0/0/120 30/0/30 40/0/20
5	41.7	67.0	95.9	0/0/120	0/0/100	0/0/100 30/0/10 15/0/10	0/0/110 30/0/10 20/0/20

*HHS* Harris Hip Score, *THA* Total hip arthroplasty, *ROM* Range of motion

## Discussion

Improvement of screening methods, in particular the introduction of ultrasound screening for developmental dysplasia of the hip, has considerably reduced the incidence of NHD in the past decades – however, in advanced stages, the condition still represents a major challenge in orthopaedics [25, 26]. Even though THA has by now become a standard procedure in orthopaedic surgery, the severe LLD and soft tissue contractures associated with unilateral NHD are difficult to address by THA alone, and can be further complicated by SSO. There is general consensus that extensive leg lengthening through THA implies an increased risk for nerve damage [17]. SSO may facilitate restoration of the centre of rotation of the hip joint and decrease the risk for nerve palsy, but there is a risk of non-union of the osteotomy site, and shortening of the femur may further contribute to LLD [15, 16]. Thus, residual LLD is a common issue after THA in unilateral NHD, and functional impairment, limping and lower back pain can negatively affect the clinical outcome [27]. While LLD of less than 2 cm can generally be handled non-operatively, this approach may be unsatisfactory in case of LLD of more than 2 cm, as shoe lifts of more than 2 cm are frequently associated with walking insecurity, apart from being cosmetically unfavourable. Regarding surgical treatment, revision arthroplasty with exchange of modular implant components may be considered in order to achieve leg lengthening. Yet this represents a major surgical intervention with increased risk for development of a periprosthetic joint infection, and complete leg length equalisation may be very difficult to achieve due to soft tissue tension [27]. Contralateral femoral shortening might be another option to attain leg length equalisation [28, 29], but may lead to irregular body proportions, reduced body height and impaired muscle strength [30]; it should thus only be taken into consideration if ipsilateral femoral lengthening is not possible, if at all.

In order to address the challenge implied by severe shortening of the soft tissues, several authors have proposed two-stage THA, with skeletal traction or iliofemoral distraction through an external fixator or distraction nail prior to THA [31–35]. However, the necessity of an excessive soft tissue release may result in functional deficiencies, and external fixation is associated with patient discomfort and pin tract infections [32, 34]; the risk for periprosthetic joint infections by contaminated pin tracks and bacteraemia might thus theoretically be increased.

Successive intramedullary femoral lengthening *after* THA, on the other hand, offers the advantage of limb length equalisation through a relatively minor surgery. In the past twenty years, intramedullary lengthening through motorised lengthening nails has become an established procedure for distraction osteogenesis [20, 30]. In particular remote-controlled magnetically-driven lengthening nails have become increasingly popular due to their easy application technique and handling [36]. Femoral lengthening by retrograde nails yielded as favourable results as femoral lengthening using antegrade nails [20].

Very few authors have yet described the concept of staged femoral lengthening for treatment of residual LLD after THA. Harkin et al. presented the results of three patients with secondary osteoarthritis of the hip joint who received THA and subsequent femoral lengthening through a PRECICE® lengthening device [21]. One of whom occurred due to Legg-Calvé-Perthes disease, and two were sequelae of septic arthritis of the hip joint without dislocation. All three patients achieved leg length equalisation and showed a satisfying functional outcome [21]. Zak et al. and Thakral et al. each described two successful cases of subsequent intramedullary femoral lengthening after THA – however, lengthening was performed at the *contralateral* femur [18, 22].

The present study shows an outcome which is similar to those of the aforementioned studies, with satisfying radiological and functional results at a reasonable complication rate [21, 22]. Nonetheless, to our knowledge this is the first study describing subsequent retrograde lengthening of the ipsilateral femur after THA with or without SSO as a treatment regimen for unilateral high hip dislocation with relevant LLD. One limitation of this study certainly is the small case number, which is owed to the rarity of the specific condition investigated, and the short follow-up period. Future investigations will have to evaluate the long-term implant survival rate. Moreover, it should be noted that ipsilateral limb lengthening subsequent to THA entails a theoretical risk of secondary dislocation of the artificial hip joint, and the requirement of additional surgeries after THA may potentially increase the risk for periprosthetic joint infections. Furthermore, limb lengthening of several centimetres implies the risk of development of an extension deficit of the knee joint, which can require compression of the distraction site.

The procedure presented in this study may be considered in case of significant LLD after THA in unilateral NHD with consecutively limited patient mobility. The treatment procedure itself is tedious and requires great patient compliance, and regular clinical check-ups are necessary to timely detect and treat any complications occurring during the lengthening and consolidation period.

## Data Availability

The datasets used and/or analysed during the current study are available from the corresponding author on reasonable request.
